# Clinical characteristic, common sites, and geographical distribution of pediatric tuberculosis patients in Southwest China

**DOI:** 10.3389/fped.2024.1327648

**Published:** 2024-03-18

**Authors:** Dong-Mei Wang, Chuan Wang, Qi An, Qing Yang, Yi Liao

**Affiliations:** ^1^ Department of Science and Education Division, Public Health Clinical Center of Chengdu, Chengdu, Sichuan, China; ^2^ Department of Clinical Laboratory Medicine, Chengdu Women’s and Children’s Central Hospital, School of Medicine, University of Electronic Science and Technology of China, Chengdu, China

**Keywords:** pediatric, tuberculosis, epidemiology, clinical characteristics, geographical distribution

## Abstract

**Background:**

The data report of a large sample, dynamic epidemiology, and characteristic analysis of pediatric tuberculosis (TB) in Southwest China is not clear. Herein, we conducted descriptive dynamic epidemiology, characteristic analysis and geographical distribution study of pediatric TB inpatients in Southwest China for more than 20 years.

**Methods:**

Patients with pediatric TB were recruited from October 2002 to September 2022 in Southwest of China based on etiology or clinical confirmation. Extract hospitalization medical record information for each patient. The geographical distribution chart of cases is used to display the trend of case flow segmented every 5 years.

**Results:**

Among 3,024 pediatric TB patients with an average age of 9.11 ± 4.39, 17.49% (529) had pulmonary tuberculosis (PTB), 9.06% (274) had extrapulmonary tuberculosis (EPTB), and 73.45% (2,221) had combined TB. The most common form of EPTB is disseminated TB (28.98%), followed by TB lymphadenitis (20.56%), pleural TB (19.72%), and TB meningitis (19.68%). Children aged 0–4 years had a high risk of TB meningitis and a severe symptoms, while children in the elderly age group had a high risk of pleural TB. In the past 20 years, hospitalized TB pediatric cases mainly came from Sichuan, Tibet, Qinghai, Yunnan and other places. The number of patients from ethnic minorities, especially Tibetans, showed an upward trend on a yearly basis (*χ*^2^ = 401.43, *P* < 0.001).

**Conclusions:**

Public health investment and effective management in pediatric TB should be further strengthened.

## Introduction

According to the latest data from World Health Organization from 2022, during 2020–2021, the incidence rate of tuberculosis (TB) increased by 3.6%, reversing the declining trend of some 2% that was observed every year in the past. Pediatric TB accounted for about 11% of global TB cases in 2021 (1). China has the second highest incidence rate of TB in the world, however, the number of pediatric TB cases reported in China is only 1% of domestic TB cases. Most of the literature reports on pediatric TB in China and worldwide were small sample sizes or limited research directions (2–6). There is a serious problem of insufficient diagnosis and reporting of pediatric TB in China and a lack of data reports on large samples of children, dynamic epidemiology, and characteristic analysis.

The prevalence of TB in children is an important basis for evaluating the effectiveness of national TB prevention and control measures, as it represents the recent or ongoing disease transmission, indirectly reflects the status of social TB infectious sources and the severity of the epidemic situation, as well as the deficiencies in TB prevention and control (7). Therefore, understanding the prevalence of pediatric TB and strengthening the prevention and control of TB is of great significance for strengthening the regional prevention and control of TB, reducing the incidence of adult secondary TB and effectively controlling TB. Herein, we conducted descriptive dynamic epidemiology, characteristic analysis and geographical distribution study of pediatric TB inpatients in Southwest China for over 20 years.

## Methods

### Study design and population

This retrospective study was based on the discharge data from the Chengdu Public Health Clinical Center (PHCC) affiliated to the central city of Sichuan Province in Southwest China. Sichuan Province is located in the Southwest hinterland of the Chinese Mainland, in the upper reaches of the Yangtze River, bordering seven provinces (autonomous regions, municipalities directly under the Central Government), including Chongqing, Guizhou, Yunnan, Tibet, Qinghai, Gansu, and Shaanxi and covering a total area of 486,000 square kilometers. Its total population in 2022 was 83.72 million. The center is the designated centralized management unit of regional TB, responsible for the prevention, control, diagnosis and treatment of TB around the area under its jurisdiction, with an annual outpatient volume of 180,000 to 200,000.

**Figure 1:**
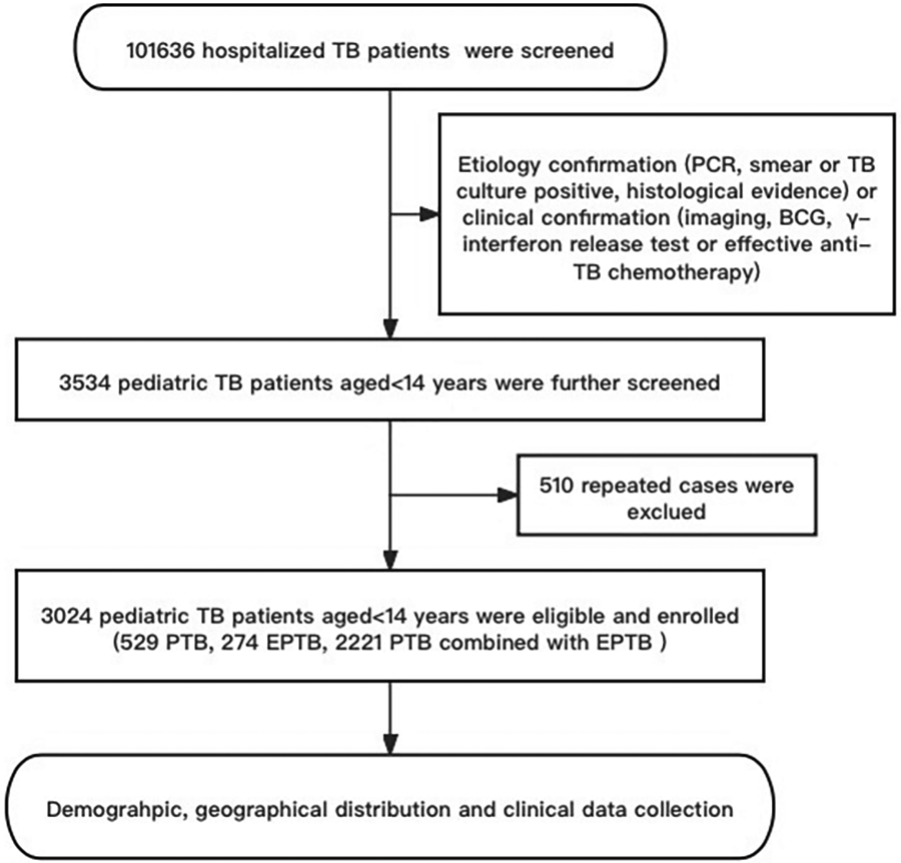
The flow diagram of our study. Demographic information and clinical data was reviewed from the Public Health Clinical Center of Chengdu, Sichuan, China.

Patients were recruited from October 2002 to September 2022 in PHCC based on etiology (cases were defined as bacteriologically confirmed when biologic specimen was positive by smear microscopy, polymerase chain reaction or culture, in accordance with WHO) or clinical confirmation [etiology does not support diagnosis, but through imaging, Bacillus Callmette-Güerin (BCG), *γ*-interferon release test or effective anti-TB chemotherapy who were diagnosed with clinical manifestations] consecutive cases. After eliminating duplicate cases, a total of 3,024 pediatric TB patients aged <14 years were included in the study. All information about these cases in the hospital medical record management system and information management system (HIS) were collected, including demography, the geographical distribution of habitual residence, clinical characteristics, outcomes, and other information (Figure 1).

### Diagnostic criteria

Diagnosis of pediatric TB was based on the Chinese Pulmonary Tuberculosis Diagnostic Criteria (WS 288–2017), the Chinese' TB volume of clinical diagnosis and treatment guidelines’ (Chinese Medical Association, 2005), and the updated WHO guidelines (1). In accordance with WHO guidelines, pulmonary tuberculosis (PTB) refers to TB disease involving the lung parenchyma and/or trachea-bronchial tree, including miliary TB with or without a diagnosis of extrapulmonary tuberculosis (EPTB), while all other sites of disease are classified as EPTB. Concurrent extra-pulmonary and pulmonary TB (combined TB) was defined as any case of TB that involved both the lungs and organs other than the lungs. Acquired immune deficiency syndrome (AIDS) was diagnosed based on the Chinese AIDS and HIV infection diagnostic criteria (WS293–2008) (8).

### Variables and outcomes

The medical record information of each patient extracted from the HIS included sociodemographic, TB-related and result variable values. Sociodemographic variables mainly included gender, age, ethnicity, and place of habitual residence. The variables related to TB included TB type, relevant diagnosis, complications, and hospital admission time. The outcome variables included treatment outcomes, length of stay and TB hospitalization cost. Information about the habitual residence was collected for all cases, then coded according to their residence (geographic code), and matched to a 1:100,000 digital map of China using Python 3.7. The cases were displayed using a geographical distribution chart of China and Sichuan Province, and the flow trend of case information was segmented every 5 years.

### Statistical analysis

SPSS Statistics Client 26.0 (SPSS Inc., IL, USA) was used for data analysis. The measurement data of normal distribution were expressed as median or mean ± standard deviation, and categorical variables were expressed as the number and percentage. Pearson *χ*^2^ was used. Multiple logistic regression models were used to analyze the correlation between various types of pediatric EPTB and age, gender, ethnicity, length of hospitalization, and disease outcomes. Linear regression was used to analyze the correlation between hospitalization expenses and age, length of hospitalization, and disease outcomes *P* < 0.05 indicated statistical significance.·

### Ethics approval and consent to participate

This study was approved by the Ethics Committee of PHCC (YJ-K2023-08-01). All patient information used in this study was routinely collected through the mandatory notification system. The requirement for informed consent was waived by the ethics committee.

## Results

### Demographic and clinical characteristics

The mean age of 3,024 pediatric TB patients was 9.11 ± 4.39 (range: 18 days–14 years) old; 20.9% of the patients were aged 0–4 years, 22.6% of the patients were aged 5–9 years, and 56.5% of the patients were aged 10–14 years. A positive correlation was observed between the age and the incidence rate over the past 20 years, with a statistically significant difference (*χ*^2^ = 47.51, *P* < 0.001). The subgroup comprised 54.8% (1,656/3,024) males and 45.2% (1,368/3,024) females, and there was no significant difference in the male-to-female ratio over 20 years (*χ*^2 ^= 0.75, *P* > 0.05) (Table 1).

**Table 1:** Demographic and clinical characteristics of persons aged ≦14 years with tuberculosis (TB) reported to the PHCC in Southwest China, 2002–2022.

Variables	2002–2007 (*n* = 78)		2008–2012 (*n* = 311)		2013–2017 (*n* = 1,194)		2018–2022 (*n* = 1,441)		*χ* ^**2**^	*p*-Value	Total (*n* = 3,024)	
	*n*	%	*n*	%	*n*	%	*n*	%			*n*	%
Sex									0.75	0.86		
Female	33	42.3	136	43.7	539	45.1	660	45.8			1,368	45.2
Male	45	57.7	175	56.3	655	54.9	781	54.2			1,656	54.8
Age									47.51	<0.001	0.0	
Mean ± SD; years (range)	6.58 ± 4.41		9.03 ± 4.20		9.38 ± 4.23		9.79 ± 3.94				9.11 ± 4.39	
0–4 (male, female)	33 (18, 15)	42.3	83 (50, 33)	26.7	272 (163, 109)	22.8	245 (151, 94)	17.0			633	20.9
5–9 (male, female)	19 (11, 8)	24.4	59 (37, 22)	19.0	266 (162, 104)	22.3	338 (190, 148)	23.5			682	22.6
10–14 (male, female)	26 (16, 10)	33.3	169 (88, 81)	54.3	656 (330, 326)	54.9	858 (440, 418)	59.5			1,709	56.5
Race/ethnicity									401.43	<0.001	0.0	
Han nationality	71	91.0	284	91.3	530	44.4	501	34.8			1,386	45.8
Tibetan	5	6.4	24	7.7	557	46.6	778	54.0			1,364	45.1
Yi	1	1.3	2	0.6	92	7.7	145	10.1			240	7.9
Qiang	1	1.3	1	0.3	12	1.0	9	0.6			23	0.8
Others	0	0.0	0	0.0	3	0.3	8	0.6			11	0.4
Co-infectious disease									200.31	<0.001	0.0	
AIDS	0	0.0	1	0.3	11	0.9	6	0.4			19	0.6
Anemia	2	2.6	45	14.5	241	20.2	304	21.1			629	20.8
Hepatitis B	0	0.0	0	0.0	16	1.3	7	0.5			24	0.8
Maligancy	0	0.0	0	0.0	2	0.2	4	0.3			6	0.2
Heart disease	1	1.3	3	1.0	8	0.7	0	0.0			15	0.5
Diseases of the immune system	0	0.0	0	0.0	0	0.0	1	0.1			1	0.0
Pulmonary infection	2	2.6	16	5.1	310	26.0	188	13.0			550	18.2
Hypoproteinemia	0	0.0	0	0.0	195	16.3	190	13.2			401	13.3
Malnutrition	6	7.7	26	8.4	121	10.1	76	5.3			255	8.4
Syphilis	0	0.0	0	0.0	1	0.1	1	0.1			2	0.1
Cerebral infarction	0	0.0	0	0.0	3	0.3	21	1.5			24	0.8
Epilepsy	3	3.8	5	1.6	50	4.2	51	3.5			119	3.9
Thyroid dysfunction	0	0.0	0	0.0	8	0.7	42	2.9			51	1.7
Length of stay									174.662	<0.001		
<7	27	34.6	55	17.7	168	14.1	143	9.9			393	13.0
7–14	16	20.5	79	25.4	313	26.2	673	46.7			1,081	35.7
>14	35	44.9	177	56.9	713	59.7	625	43.4			1,550	51.3
Disease outcome									248.262	<0.001		
Get better	58	74.4	295	94.9	1,164	97.5	1,408	97.7			2,925	96.7
Cure	1	1.3	10	3.2	18	1.5	8	0.6			37	1.2
Unhealed	12	15.4	4	1.3	4	0.3	10	0.7			30	1.0
Death	4	5.1	1	0.3	6	0.5	3	0.2			14	0.5
Others^a^	3	3.8	1	0.3	2	0.2	12	0.8			18	0.6
Adverse drug reaction									93.71	<0.001		
Liver dysfunction	4	5.1	28	9.0	159	13.3	193	13.4			411	13.6
Rash	0	0.0	1	0.3	7	0.6	10	0.7			19	0.6
Hyperuricemia	1	1.3	31	10.0	328	27.5	574	39.8			973	32.2
Leucopenia	0	0.0	12	3.9	5	0.4	17	1.2			38	1.3
Thrombocytopenia	0	0.0	2	0.6	25	2.1	22	1.5			52	1.7

PHCC, Public Health Clinical Center of Chengdu.

^a^ Unexpected conditions of discharges; The ATD-induced hepatotoxicity is defined as ALT ≥3 ULN. Hemoglobin <110 g/L, 5–11.99 years. Hemoglobin <115 g/L, 12–14.99 years. Hemoglobin <120 g/L (9).

In 20 years, the number of Han pediatric TB patients showed a downward trend every year, while the number of ethnic minorities, especially Tibetan patients, showed an upward trend year by year, with a statistically significant difference (*χ*^2 ^= 401.43, *P* < 0.001). The top three comorbidities among the study cases were: anemia (629, 20.8%), pulmonary infection (550, 18.2%), and hypoproteinimia (401, 13.3%). The average length of stay for a patient was 17.73 days. Furthermore, 2,925 patients (96.7%) improved after discharge. Among the study cases, 973 (32.2%) had hyperuricemia, 411 (13.6%) had liver dysfunction, 22 (1.5%) had thrombocytopenia, 17 (1.2%) had leucopenia, and 10 (0.7%) had rash due to adverse reactions caused by anti-tuberculosis drugs (Table 1).

### Type distribution and age proportion among PTB, EPTB, and combined TB groups

**Figure 2:**
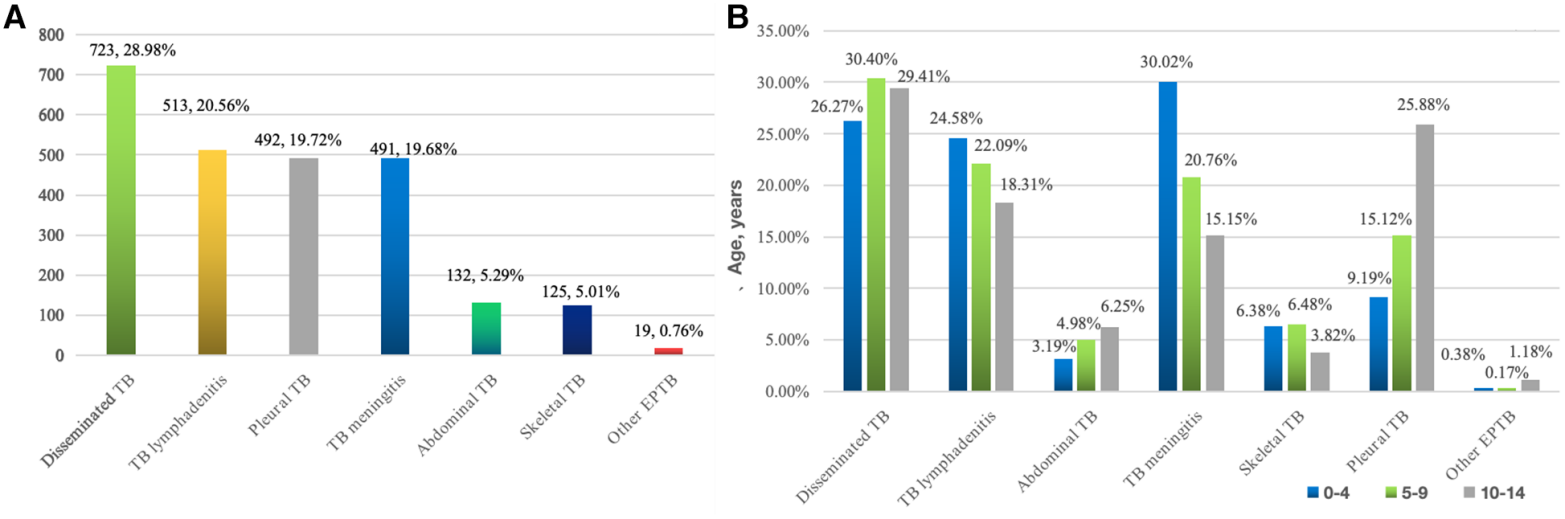
(**A**) Location distribution of 2,495 pediatric EPTB * (676 cases of disseminated TB involved 2 extrapulmonary lesions, 35 cases at 3 sites, 10 cases at 4 sites, and 2 cases at 5 sites). (**B**) Age distribution of different parts of tuberculosis in children.

Among these patients, 17.49% (529) had PTB, 9.06% (274) had EPTB, and 73.45% (2,221) had combined TB. The most common form of pediatric EPTB infection was disseminated TB (*n* = 723, 28.98%), while the other forms included lymphatic TB (*n* = 513, 20.56%), pleural TB (*n* = 492, 19.72%), TB meningitis (*n* = 491, 19.68%), abdominal TB (*n* = 132, 5.29%), skeletal TB (*n* = 125, 5.01%) and other EPTB (*n* = 19, 0.76%) (Figure 2A).

The composition ratio of EPTB types in various age groups shows that the proportion of TB meningitis (30.02%), disseminated TB (26.27%), and lymphatic TB (24.58%) in children aged 0–4 years was relatively high; disseminated TB (30.40%), lymphatic TB (22.09%), and TB meningitis (20.76%) were relatively high in those aged 5–9 years, while disseminated TB (29.41%), pleural TB (25.88%), and lymphatic TB (18.31%) were relatively high among the 10–14-year-old patients (Figure 2B).

Next, we analyzed the correlation between the type of pediatric EPTB between gender, age, ethnicity, length of stay, and disease outcome using a multivariate logical model. The results showed that in terms of age, the risk of TB meningitis was higher (OR = 2.471, 95% CI: 1.930–3.163) between 0 and 4 years patients compared to 10–14 years patients, the following were lymphatic TB (OR = 1.504, 95% CI: 1.172–1.932) and skeletal TB (OR = 1.744,95% CI: 1.102–2.759). At 5–9 years of age, the risk of pleural TB was high (OR = 1.875, 95% CI: 1.290–2.725), while the risk of TB meningitis was relatively low (OR = 0.601, 95% CI: 0.456–0.792); 10–14 years patients also had the highest risk of pleural TB (OR = 3.745, 95% CI: 2.689–5.198), followed by dominant TB (OR = 2.123, 95% CI: 1.223–3.687), while they had a low risk of TB meningitis (OR = 0.405, 95% CI: 0.316–0.518), skeletal TB (OR = 0.573, 95% CI: 0.362–0.907), and lymphatic TB (OR = 0.665, 95% CI: 0.518–0.853).

In terms of ethnic distribution, Qiang people had a higher risk of TB meningitis compared to Han people (OR = 2.982,95% CI: 1.001–8.880). However, the location of pediatric EPTB was not related to gender and length of stay (Table 2).

**Table 2:** Multivariate analysis of associated factors for different forms of EPTB, Southwest of China, 2002–2022.

Characteristic	Adjusted odds ratio (95% CI)						
	TB lymphadenitis	Abdominal TB	TB meningitis	Skeletal TB	Pleural TB	Other EPTB	Disseminated TB
	1	2	3	4	5	6	7
Sex							
Boy	Referent	Referent	Referent	Referent	Referent	Referent	Referent
Girl	0.926 (0.76, 1.129)	1.101 (0.771, 1.57)	1.003 (0.819, 1.228)	0.949 (0.658, 1.37)	0.953 (0.778, 1.168)	0.788 (0.314, 1.978)	1.087 (0.912, 1.296)
Age, year							
0–4	Referent	Referent	Referent	Referent	Referent	Referent	Referent
5–9	0.846 (0.639, 1.12)	1.651 (0.886, 3.075)	**0.601 (0.456, 0.792)**	1.032 (0.636, 1.676)	**1.875 (1.29, 2.725)**	0.443 (0.039, 4.968)	1.204 (0.925, 1.568)
10–14	**0.665 (0.518, 0.853)**	**2.123 (1.223, 3.687)**	**0.405 (0.316, 0.518)**	**0.573 (0.362, 0.907)**	**3.745 (2.698, 5.198)**	3.223 (0.707, 14.685)	1.153 (0.913, 1.455)
Race/ethnicity							
Han nationality	Referent	Referent	Referent	Referent	Referent	Referent	Referent
Tibetan	0.992 (0.807, 1.219)	1.166 (0.801, 1.696)	1.141 (0.924, 1.411)	0.988 (0.672, 1.452)	0.822 (0.664, 1.018)	2.048 (0.772, 5.433)	0.99 (0.823, 1.191)
Yi	0.852 (0.587, 1.236)	0.894 (0.432, 1.852)	0.779 (0.528, 1.151)	1.007 (0.528, 1.922)	1.234 (0.859, 1.772)	—	1.206 (0.881, 1.652)
Qiang	1.11 (0.309, 3.984)	2.557 (0.557, 11.731)	**2.982 (1.001, 8.88)**	1.796 (0.229, 14.087)	0.412 (0.092, 1.844)	—	0.362 (0.081, 1.62)
Other	0.316 (0.04, 2.473)	2.213 (0.275, 17.824)	0.653 (0.139, 3.068)	—	1.673 (0.423, 6.619)	—	1.862 (0.584, 5.94)
Length of stay(LOS), d							
<7	Referent	Referent	Referent	Referent	Referent	Referent	Referent
7–14	1.013 (0.729, 1.407)	1.113 (0.602, 2.056)	0.967 (0.699, 1.339)	1.182 (0.637, 2.195)	0.769 (0.542, 1.092)	0.404 (0.093, 1.755)	1.186 (0.875, 1.607)
>14	1.035 (0.754, 1.421)	0.909 (0.499, 1.657)	0.899 (0.657, 1.23)	1.202 (0.664, 2.177)	0.851 (0.609, 1.188)	0.502 (0.133, 1.893)	1.189 (0.886, 1.595)
Discharge							
Leave the hospital on doctor's order	Referent	Referent	Referent	Referent	Referent	Referent	Referent
Discharged without a doctor's advice	0.30 (0.072, 1.312)	**4.048 (1.343, 12.205)**	1.114 (0.435, 2.848)	**4.652 (1.693, 12.785)**	0.706 (0.234, 2.127)	—	0.616 (0.23, 1.646)
Cure	**3.637 (1.639, 8.068)**	1.579 (0.364, 6.844)	0.598 (0.176, 2.026)	—	0.888 (0.326, 2.419)	—	0.343 (0.102, 1.151)
Death	0.351 (0.045, 2.733)	—	1.393 (0.369, 5.262)	—	0.372 (0.047, 2.943)	—	**3.267 (1.031, 10.356)**
Other^a^	1.275 (0.334, 4.861)	**8.7 (2.195, 34.478)**	1.154 (0.299, 4.453)	—	0.59 (0.074, 4.714)	—	0.261 (0.033, 2.054)

^a^ Unexpected conditions of discharges.

The bold values means the difference is statistically significant with *P* < 0.05.

### Geographical distribution

**Figure 3:**
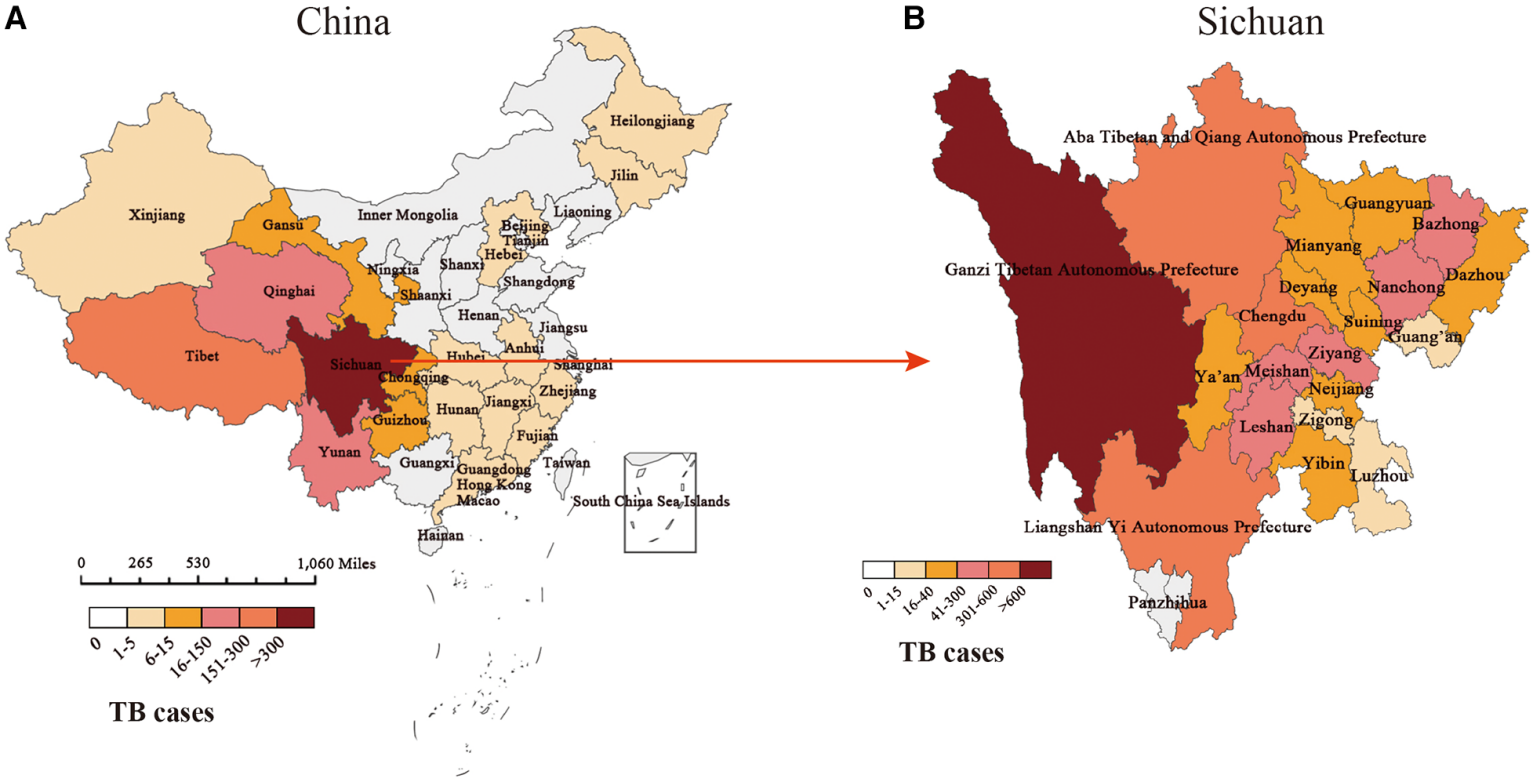
Distribution of pediatric TB cases in our study. (**A**) The geographical distribution of pediatric TB cases in China. (**B**) The geographical distribution of pediatric TB cases in Sichuan.

Among 3,024 pediatric TB patients diagnosed and treated in PHCC between October 2002 and September 2022, 53 had missing information about their usual places of residence, and the rest were matched to a 1:100,000 digital map of China using Python 3.7. According to the geographical distribution map, the geographical distribution sources of 2,971 pediatric TB cases from 2002 to 2022 covered more than two-thirds of China's regions. With Sichuan Province as the center, all cities in Southwest China Chongqing, Guizhou, Yunnan and Tibet, were found to have case sources, with Tibet having the largest case distribution (151–300). Xinjiang, Qinghai, and Gansu resulted as the main source areas of cases in the northwest region, especially in Qinghai (16–150). Central South and East China cases were mainly distributed in Hebei, Anhui, Hunan, Jiangxi, Zhejiang, Fujian, Guangdong, Hong Kong, and Macao regions, with relatively small case distribution (1–5). The cases in North China and Northeast China were mainly in Heilongjiang, Jilin, Beijing, Tianjin, and Hebei, and the distribution of these cases was relatively small (1–5) (Figure 3).

**Figure 4:**
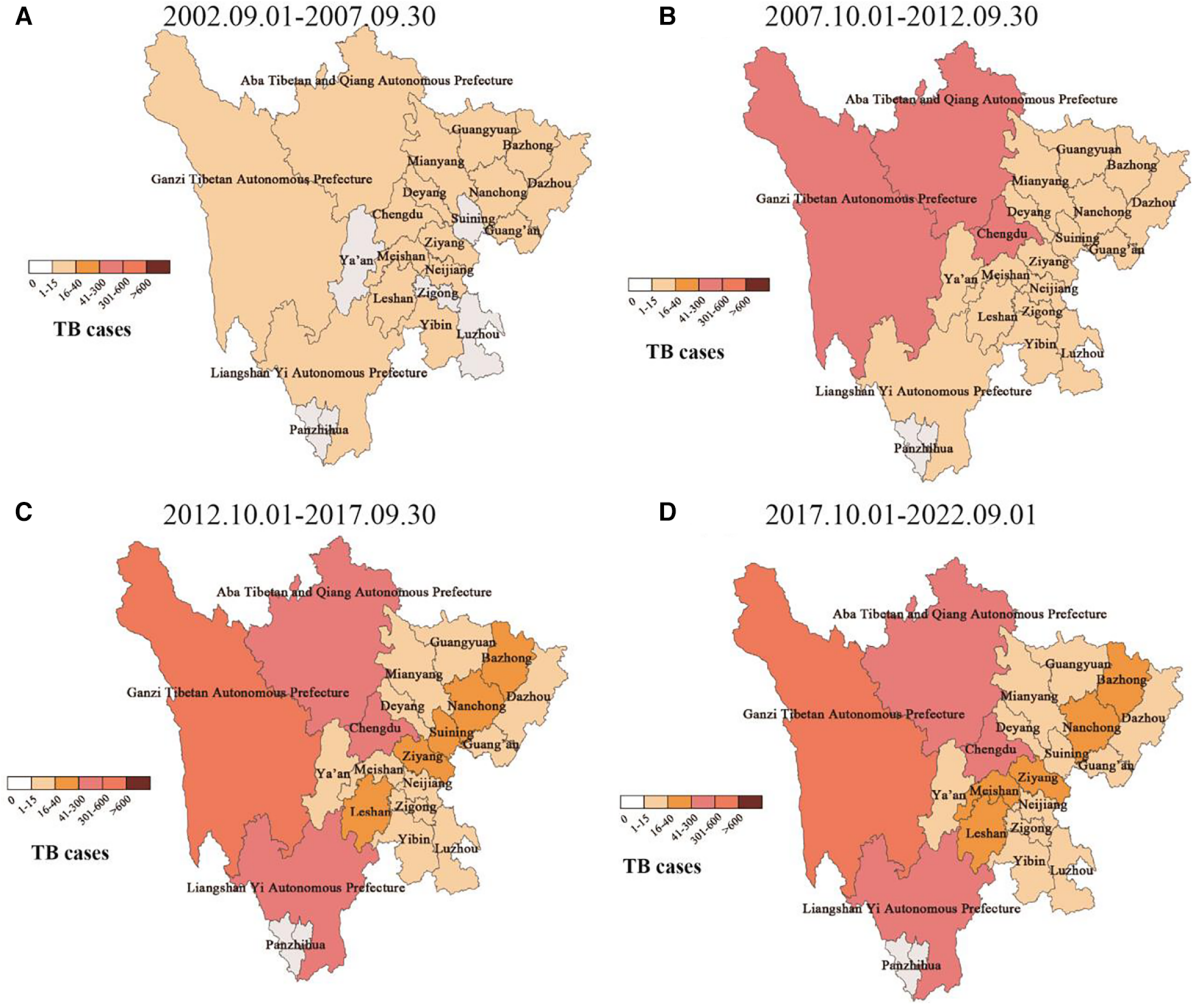
Dynamic geographical distribution of pediatric TB cases in sichuan province. (**A**–**D**) Represents the geographical distribution of case sources in different years.

The distribution of cases in the central city of this study, Sichuan Province, is mainly in minority areas such as Ganzi Tibetan Autonomous Prefecture (>600), Aba Tibetan and Yi Autonomous Prefecture (301–600), and Liangshan Yi Autonomous Prefecture (301–600) in western Sichuan. The segmented case trend chart over the past 20 years showed that the source of cases gradually spread from the initial territory of Sichuan Province, China, to Southwest, northwest, central, eastern, and northern regions of China, but Sichuan, Qinghai, Tibet, and Yunnan in Southwest China had the high incidence areas of pediatric TB. Among the ethnic minority areas in western Sichuan, the segmented case trend chart in Sichuan showed that regions such as Ganzi, Aba, and Liangshan, were high-incidence areas of pediatric TB (Figure 4).

## Discussion

In the present study, we dynamically described the epidemiological characteristics of pediatric TB patients in Southwest China based on the big data analysis covering more than 20 years of cases diagnosed and treated in PHCC, the designated centralized hospital for TB in Chengdu, the most representative central city in Southwest China. To the best of our knowledge, this is the most representative study of high-incidence areas of TB in China, which included the hospitalized pediatric TB patients with the largest number of cases and the longest time span. This is also the first study that proved the serious prevalence of pediatric TB in the high-incidence area of TB in Southwest China over the past 20 years, elucidating the trends of epidemic change.

In the present study, the incidence of pediatric TB in Southwest China was dominated by boys, and the proportion of boys and girls was basically the same as that of adults (10). A total of 82.51% of children were found to have EPTB or combined TB, which is much higher than in other regions in China (46%) (11), including Beijing (54%) (12) and Shandong (73%) (13), the United States (24.52%) (14), the United Kingdom (38.11%) (15), India (17.68%) (16), and other places.

In addition, our data showed that disseminated TB was the main type of infection among hospitalized pediatric TB patients, accounting for 28.98% of all EPTB and combined TB cases. These results are not consistent with the previous domestic study of EPTB in children, where TB meningitis (34.18%) was the main type of infection, or a domestic study in Shandong Province, where pleural TB (29.0%) was the main type of infection. In this study, TB meningitis (32.59%) was mainly concentrated in young children and was the main type of infection among hospitalized children <4 years, while pleural TB (71.54%) and abdominal TB (64.39%) were the main types of infection among hospitalized children in the middle and elderly age groups. At the same time, TB meningitis and disseminated TB led to the highest hospitalization expenses among different types of cases. Furthermore, the hospitalization expenses were positively correlated with the length of stay and patient's age, while negatively correlated with the length of stay, death, and other outcomes. It was further suggested that pediatric TB, especially in young children, was associated with a high risk of TB meningitis, with rapid disease development and a more severe condition. Therefore, clinical attention should be further strengthened, and early control should be taken.

With the increasing global concern about the prevalence of pediatric TB and the high burden of malnutrition worldwide, it has been increasingly recognized that malnourished children are the main high-risk group of TB (17, 18). The conflict between these two diseases is key to improving children's health. In this study, a large proportion of pediatric TB patients had anemia (20.8%), hypoalbuminemia (13.3%) and malnutrition (8.4%). Relevant research shows that nutrition intervention can significantly improve the cure rate of TB (19). Therefore, while carrying out targeted TB case detection, nutritional support and treatment for malnourished children in key areas should be strengthened to improve their nutritional status.

With the incidence rate of HIV/AIDS rising year by year, the HIV/MTB double infection rate among children has also been increasing (20–23). In this study, 19 cases (0.6%) had HIV infection. Most of the combined AIDS cases came from high AIDS incidence areas such as Liangshan Yi Autonomous Prefecture and Aba, while some cases came from the surrounding areas of Chengdu. The minimal case age was 1 year old, and the maximal case age was 12. MTB combined with HIV infection aggravated the progress of the two diseases (1, 23). Therefore, while strengthening the detection of pediatric TB, especially in areas with a high incidence of AIDS, HIV infection should be screened as soon as possible and standardized to determine whether AIDS is combined. Also, timely treatment should be carried out to control TB effectively.

A 4-year retrospective study on pediatric TB showed that neighboring Qinghai and Kunming had a high incidence rate of pediatric TB in China; however, the research cycle of this study was short, and data were missing for key areas such as Sichuan, Chongqing and Tibet (6). In this study, the dynamic epidemiological characteristics and trends of pediatric TB in Southwest China were analyzed by segments including data for >20 years, which further confirmed the serious prevalence of pediatric TB in Sichuan, Tibet, Qinghai, and other places in China, mainly concentrated in Ganzi, Aba, Liangshan, and other ethnic minority areas in Sichuan Province. In this study, 54.2% of pediatric TB were ethnic minorities, among which the Tibetan population accounted for the largest proportion. At the same time, the proportion of cases from ethnic minorities has significantly increased from 9.0% in the previous five years to 65.2% in the recent five years, while the proportion of cases from the Han population has shown a downward trend from 91.0% in the previous five years to 34.8% in the recent five years. In our previous study of TB meningitis in children, we found that the proportion of ethnic minority patients was very high (81%), and the coverage rate of the BCG vaccine was very low (24).

Although the Chinese government made significant efforts to prevent and control TB in Southwest China in recent years, which were successful to a certain extent, the prevention and control of TB are still poor due to the remote geographical location of Tibet, Qinghai, Ganzi, Aba, Liangshan, and other places in Southwest China, the relatively backward medical conditions, the problems communication, and the poor awareness of BCG vaccination and disease medical care. Accordingly, the government should continue to encourage relevant prevention and control departments to formulate targeted strategies to strengthen the prevention and control of TB in Southwest China, especially in ethnic minority areas, in order to further eliminate TB.

In conclusion, in Southwest China, hospitalized pediatric TB mainly concentrated in ethnic minority areas in western Sichuan and other places outside the province, such as Tibet, Qinghai and Yunnan in the past 20 years. The most common type of EPTB among children was disseminated TB, and the risk of TB meningitis in young children was high and severe. Therefore, publicity, education, and policy support in pediatric TB should be increased, especially in ethnic minority areas in Southwest China.

## Data Availability

The raw data supporting the conclusions of this article will be made available by the authors, without undue reservation.
